# PD-1 Blockade in Chronically HIV-1-Infected Humanized Mice Suppresses Viral Loads

**DOI:** 10.1371/journal.pone.0077780

**Published:** 2013-10-21

**Authors:** Edward Seung, Timothy E. Dudek, Todd M. Allen, Gordon J. Freeman, Andrew D. Luster, Andrew M. Tager

**Affiliations:** 1 Center for Immunology and Inflammatory Diseases, Massachusetts General Hospital and Harvard Medical School, Charlestown, Massachusetts, United States of America; 2 Division of Rheumatology, Allergy and Immunology, Massachusetts General Hospital and Harvard Medical School, Charlestown, Massachusetts, United States of America; 3 Pulmonary and Critical Care Unit, Massachusetts General Hospital and Harvard Medical School, Charlestown, Massachusetts, United States of America; 4 Ragon Institute of MGH, Massachusetts Institutes of Technology, and Harvard, Cambridge, Massachusetts, United States of America; 5 Department of Medical Oncology, Dana-Farber Cancer Institute, Department of Medicine, Harvard Medical School, Boston, Massachusetts, United States of America; University of Pittsburgh Center for Vaccine Research, United States of America

## Abstract

An estimated 34 million people are living with HIV worldwide (UNAIDS, 2012), with the number of infected persons rising every year. Increases in HIV prevalence have resulted not only from new infections, but also from increases in the survival of HIV-infected persons produced by effective anti-retroviral therapies. Augmentation of anti-viral immune responses may be able to further increase the survival of HIV-infected persons. One strategy to augment these responses is to reinvigorate exhausted anti-HIV immune cells present in chronically infected persons. The PD-1-PD-L1 pathway has been implicated in the exhaustion of virus-specific T cells during chronic HIV infection. Inhibition of PD-1 signaling using blocking anti-PD-1 antibodies has been shown to reduce simian immunodeficiency virus (SIV) loads in monkeys. We now show that PD-1 blockade can improve control of HIV replication *in vivo* in an animal model. BLT (Bone marrow-Liver-Thymus) humanized mice chronically infected with HIV-1 were treated with an anti-PD-1 antibody over a 10-day period. The PD-1 blockade resulted in a very significant 45-fold reduction in HIV viral loads in humanized mice with high CD8^+^ T cell expression of PD-1, compared to controls at 4 weeks post-treatment. The anti-PD-1 antibody treatment also resulted in a significant increase in CD8^+^ T cells. PD-1 blockade did not affect T cell expression of other inhibitory receptors co-expressed with PD-1, including CD244, CD160 and LAG-3, and did not appear to affect virus-specific humoral immune responses. These data demonstrate that inhibiting PD-1 signaling can reduce HIV viral loads *in vivo* in the humanized BLT mouse model, suggesting that blockade of the PD-1-PD-L1 pathway may have therapeutic potential in the treatment of patients already infected with the AIDS virus.

## Introduction

Antiviral T cells play a pivotal role in the control of viremia during acute and chronic Human Immunodeficiency Virus (HIV) infection. Compelling data show that CD8^+^ T cell responses are a major component of human immune response associated with the precipitous decline from peak viremia during acute HIV infection [1], [2], [3]. These CD8^+^ T cells can inhibit HIV replication *in vitro* [4], and experimental depletion of CD8^+^ T cells in non-human primates infected with SIV abrogated their inability to contain peak viremia in acute infection, and increased viremia during chronic infection [5]. In addition to cytotoxicity to infected cells [6], effective CD8^+^ T cells may control HIV replication through a number of other mechanisms, including the release of soluble factors such as CCR5 chemokine ligands capable of inhibiting HIV replication [4], [7], [8], [9], [10], [11].

Despite the apparent ability of immune responses to restrain HIV viremia to a relatively stable plateau during the prolonged phase of chronic infection, progression to AIDS ultimately ensues in most HIV-infected persons, accompanied by dramatic increases in levels of viremia. In contrast to the high functional capacity of effector and memory CD8^+^ T cells generated after acute viral infection, CD8^+^ T cell function is often impaired or exhausted during chronic infections [12]. T cell exhaustion was originally described during chronic lymphocytic choriomeningitis virus (LCMV) infection in mice in which virus-specific CD8^+^ T cells persisted indefinitely but had reduced capacity to kill infected cells or secrete antiviral cytokines [12]. Primate and human studies have demonstrated the presence of dysfunctional CD8^+^ T cells during chronic infections with SIV in primates, as well as chronic HIV, hepatitis B, hepatitis C, and human T lymphotropic virus (HTLV) infections in humans [13].

Programmed Death 1 (PD-1, CD279) is highly expressed on exhausted CD8^+^ T cells in chronic LCMV infected mice [14]. Inhibiting PD-1 signaling *in vivo* using mAbs to either PD-1 itself or its ligand PD-L1 during chronic LCMV infection dramatically enhanced virus-specific T cell number and function leading to a marked reduction in viral load [14]. The PD-1-PD-L1 pathway was subsequently found to play a major role in CD8^+^ T cell dysfunction in chronic HIV infection in humans [15], [16], [17]. PD-1 is highly expressed on exhausted HIV-specific CD8^+^ T cells, and its levels correlate with measures of disease severity, such as viral load and declining CD4 count. Blockade of the pathway *ex vivo* with mAbs to PD-1 or PD-L1 leads to increased HIV-specific CD8^+^ T cell proliferation and production of IFNγ, TNFα, and granzyme B, indicating an overall increase in effector function [15], [16], [17]. Recently, *in vivo* blockade of the PD-1-PD-L1 pathway using anti-PD-1 mAb in chronic SIV-infected macaques resulted in rapid expansion of virus-specific CD8^+^ T cells with improved effector function [18]. Most importantly, the blockade was associated with significant reduction in viral load and prolonged survival of the SIV-infected macaques.

The limited species tropism of the HIV virus has made it very difficult to study in animal models. In efforts to “humanize” mice to render them permissive for HIV infection, investigators began to engraft human immune cells and/or tissues into immunodeficient mice that are unable to reject xenogeneic grafts [19]. Early versions of humanized mice used for HIV investigation were generated by transfer of mature human peripheral blood lymphocytes into mice homozygous for the severe combine immune deficiency (scid) mutation (Hu-PBL-scid mice) [20], or transplantation of fetal human thymus and liver tissues into scid mice (SCID-Hu mice) [21]. These mice are able to support productive HIV infection *in vivo* [22], [23] and have provided investigators with useful models of HIV infection for some applications, but have important limitations. These mice generally lack robust primary adaptive immune responses, thus limiting their usefulness for studying anti-HIV immune responses and mechanisms by which the virus evades these responses [24]. However, recent improvements in humanized mouse models of HIV infection have generated mice that are able to generate robust cellular HIV-specific responses *in vivo*. One of the most successful of the recently developed models is the BLT (Bone Marrow-Liver-Thymus) humanized mouse, which is generated by surgically implanting human fetal thymic and liver tissue into immunodeficient mice concurrently with the transfer of human hematopoietic stem cells [25], [26], [27]. Importantly in this model, human T cells are educated by autologous human thymic tissue. BLT mice demonstrate robust repopulation of mouse mucosal tissues with human immune cells that support rectal and vaginal transmission of HIV [28], [29], and robust repopulation of mouse lymphoid tissues with functional human T lymphocytes [26], [27], [30] able to force the evolution of HIV “escape mutations” [31].

We previously reported human T cells in chronically HIV-infected BLT mice demonstrate increased PD-1 expression, and that T cell PD-1 levels in these mice correlate positively with viral loads and inversely with CD4^+^ cell levels, as seen in human infection [25]. Here we demonstrate that substantial CD8^+^ T cell upregulation of PD-1 occurs in most, but not all, chronically HIV-infected BLT mice, and that PD-1 mAb treatment significantly reduces viral load specifically in those mice with high CD8^+^PD-1^+^ cells. Without PD-1 blockade, control mice maintained peak viral loads for months after HIV infection, despite our previous demonstration that acute CD8^+^ T cell responses in these mice are similar to humans in terms of specificity, kinetics, and dominant targets [31].

## Materials and Methods

### BLT humanized mice

NOD/SCID/IL2Rγ_c_^−/−^ (NSG) mice (The Jackson Laboratory) were housed in a pathogen-free facility at Massachusetts General Hospital, maintained in microisolator cages, fed autoclaved food and water, and reconstituted with human tissue as previously described [25]. Briefly, sublethally irradiated NSG mice received 1 mm^3^ fragments of human fetal liver and thymus that were implanted under one kidney capsule, and 5×10^4^–1×10^5^ purified autologous CD34^+^ hematopoietic stem cells isolated from the fetal liver were injected intravenously. After 14–18 weeks, healthy mice that met the following criteria for adequate human reconstitution were used in experiments: (1) >25% of peripheral blood cells were within a lymphocyte gate on forward-versus-side scatter plots; (2) >50% of cells in the lymphocyte gate were human (hCD45^+^/mCD45^−^); and (3) >40% of human cells in the lymphocyte gate were T cells (hCD3^+^). Two different human fetal donors were used to generate the BLT mice used in this study.

### Ethics Statement

This study was carried out in strict accordance with the recommendations contained in the Guide for the Care and Use of Laboratory Animals of the National Institutes of Health. All protocols were approved by the Subcommittee on Research and Animal Care (SRAC), which serves as the Institutional Animal Care and Use Committee (IACUC) for Massachusetts General Hospital (Protocol # 2009N000136).

### HIV infections

Viral stocks of the R5-tropic HIV-1 molecular clone JR-CSF were produced through transfection of human embryonic kidney (HEK) 293T cells, and titered as described [32]. Mice were infected intraperitoneally with 5×10^4^ TCID_50_ of JR-CSF HIV-1. Every 2–4 weeks after infection, approximately 200 µl of blood was obtained through puncture of the retro-orbital sinus for isolation of plasma virus.

### RNA isolation and viral load measurement

Viral RNA was isolated from plasma samples with the QIAamp Viral RNA Mini Kit (Qiagen). Plasma viral loads were determined by quantitative RT-PCR with the QuantiFast SYBR Green RT-PCR kit (Qiagen) as described [32].

### *In vivo* antibody treatment

BLT mice were injected with either a partially humanized mouse anti-human PD-1 mAb (clone EH12-1540-29C9) or a control mAb (SYNAGIS). This anti-PD-1 mAb has mouse variable heavy chain domain linked to human IgG1 (mutated to reduce FcR and complement binding) and mouse variable light chain domain linked to human Kappa. This anti-PD-1 mAb has been shown to bind to human PD-1 and block interactions between PD-1 and its ligands [18], [33]. SYNAGIS is a humanized mouse monoclonal antibody (IgG1κ) specific to F protein of respiratory syncytial virus (RSV) (Medimmune, Gaithersberg, MD). Antibodies (200 µg/dose) were administered intraperitoneally at on days 0, 3, 7 and 10. The dosage and schedule were based on prior *in vivo* administration of these antibodies in macaques infected with SIV [18].

### Flow Cytometry

PBMCs obtained from BLT mice were stained and analyzed using an LSRII flow cytometer (BD Biosciences). Fluorescently labeled anti-human CD45, CD4, CD8, CD244, CD160, and PD-1 Abs were obtained from BioLegend (San Diego, CA). Fluorescently labeled anti-human LAG-3 Ab was obtained from R&D Systems.

### Western Blotting

HIV-specific IgM and IgG human antibodies were detected in plasma samples from HIV-infected BLT mice using Genetic Systems (GS) HIV-1 Western Blot kits (Bio-Rad) according to the manufacturer's instructions, substituting mouse anti-human IgM and anti-human IgG antibodies conjugated to horseradish peroxidase (Southern Biotech, AL) for the anti-human Ig antibody supplied. Antibodies were detected in a final dilution of mouse plasma of 1∶101, the same dilution as that recommended by the manufacturer for the detection of HIV-specific antibodies in human clinical samples. The Western Blots were developed with ECL Plus Western blotting detection reagents (GE Healthcare).

### ELISAs

ELISAs to determine titers of IgG antibodies binding to p24 and gp120 were performed as follows. Microtiter plates (Nunc MaxiSorp, Thermo Scientific) were coated with 0.25 µg/ml recombinant p24 (HXBc2) or gp120 (JRCSF) (Immune Technology Corp, NY) overnight at 4°C. Plates were blocked with 5% bovine serum albumin (BSA) before being incubated with serial dilutions of plasma samples or HIVIG control (NIH AIDS Reagent Program) in phosphate-buffered saline (PBS), for 1.5 hours at room temperature. Antibody binding was detected with HRP-labeled anti-human IgG monoclonal antibody (1∶1,000; Southern Biotech) and a TMB peroxidase substrate (KPL, MD).

### Statistical analysis

Mann-Whitney tests or Wilcoxon matched pairs tests were determined using Prism software (GraphPad Software, Inc.) to assess statistical significance. All tests were two tailed, and *P*<0.05 was considered significant.

## Results

### PD-1 expression on CD8^+^ T cells increased in chronic HIV-1 infection

**Figure 1:**
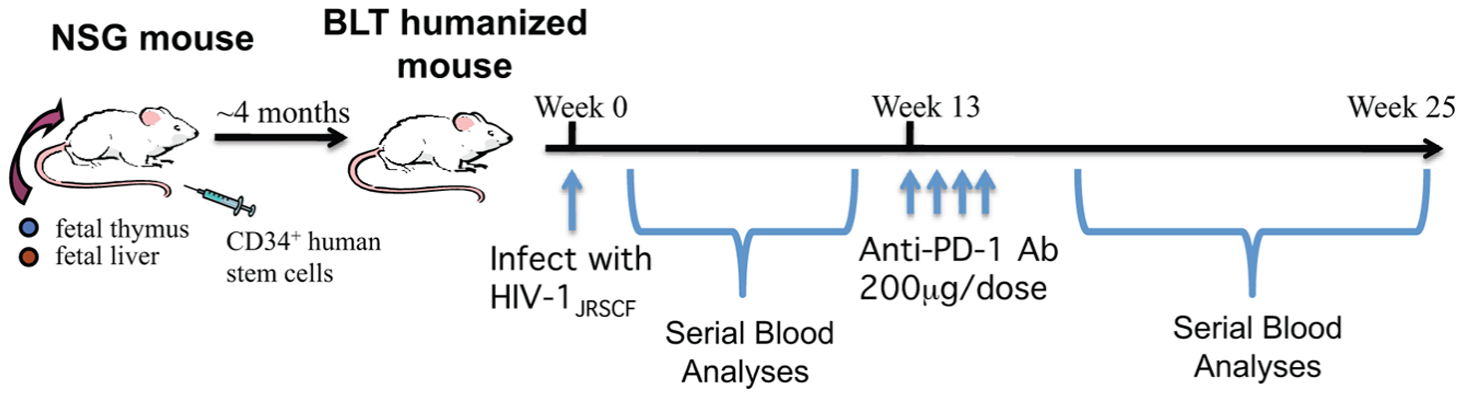
Schematic of BLT humanized mouse generation and timeline of HIV-1 infection and anti-PD-1 mAb treatment.

**Figure 2:**
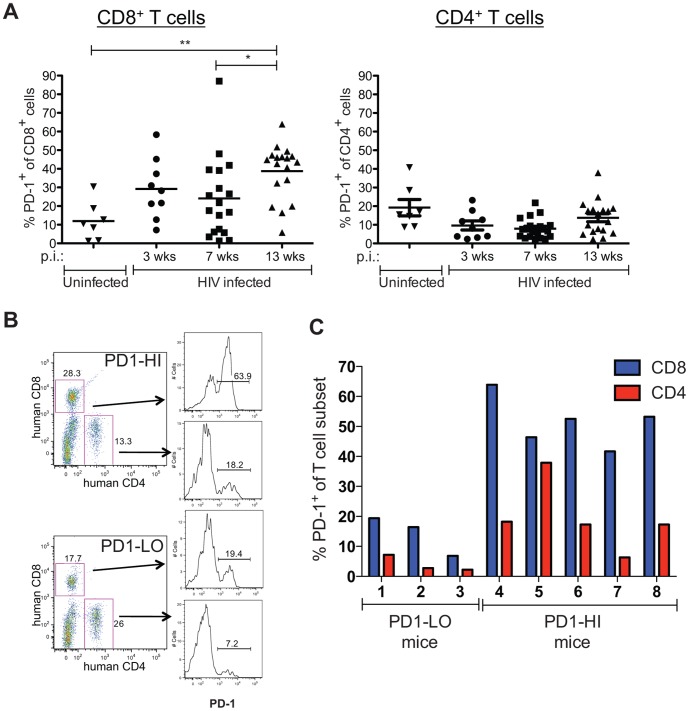
PD-1 expression on T cells in HIV-infected BLT mice. Peripheral blood was obtained at various time points after HIV infection. **A**) Percentages of human CD8^+^ and CD4^+^ T cells expressing PD-1. Horizontal lines within data points depict mean value. Uninfected controls were from peripheral blood samples obtained at time points when littermates were infected for 9-13 weeks. (HIV infected: n = 9 mice at wk 3, n = 18 mice at other time points; Uninfected: n = 7 mice). *P = 0.01, Wilcoxon matched pairs test; **P = 0.001, Mann-Whitney test. **B**) Representative flow cytometry data of PD1 expression on CD8^+^ or CD4^+^ T cells at 13 weeks post infection. PD1-HI representative is mouse #4 and PD1-LO is mouse #1 depicted in the next panel. **C**) Percentages of CD8+ and CD4+ T cells expressing PD-1 in PD1-LO (defined as having <30% PD-1^+^CD8^+^ cells) and PD1-HI (>30% PD-1^+^CD8^+^ cells).

Humanized BLT mice were created as previously described [25] with all experimental mice having met the criteria for adequate human reconstitution outlined in the Materials and Methods section. BLT mice were infected with HIV-1 virus on Week 0, as depicted in Figure 1. Peripheral blood samples were obtained serially every few weeks after infection for analyses. Infected BLT mice showed a significant increase in the percentage of CD8^+^ cells expressing PD-1 at 13 weeks post infection (p.i.) when compared to an earlier time point at 7 weeks p.i. or to uninfected BLT mice (Figure 2A). At 13 weeks p.i., 38.7±14.7% of CD8^+^ T cells expressed PD-1 on their surface, which is 1.6-fold more than at 7 weeks p.i. (24.1±21.6%) and 3.2-fold more than in uninfected controls (11.9±10.2%). In contrast, PD-1 expression on CD4^+^ T cells did not significantly increase in HIV-infected BLT mice at 13 weeks p.i. compared to earlier time points p.i. or to uninfected mice.

### PD-1 expression on CD8^+^ T cells varied among chronically HIV-infected BLT mice

Although the majority of the HIV-infected BLT mice demonstrated a dramatic increase in PD-1 expression on their CD8^+^ T cells, some mice did not. Different extents of CD8^+^ T cell PD-1 expression observed at 13 weeks p.i. are shown for two HIV-infected mice in Figure 2B: 63.9% of the CD8^+^ T cells of the mouse designated “PD1-HI” were PD-1^+^ (as were 18.2% of the CD4^+^ T cells of this mouse), whereas 19.4% of the CD8^+^ T cells (and 7.2% of the CD4^+^ T cells) of the “PD1-LO” mouse expressed PD-1. These differences in PD-1 expression occurred despite these two mice having similar HIV viral loads at this time point: the PD1-HI mouse had 2.05×10^6^ copies of HIV/mL, whereas the PD1-LO mouse had 2.42×10^6^ copies/mL. In the 18 HIV-infected mice evaluated at 13 weeks p.i., PD-1 expression ranged from a high of 63.9% to a low of 5.8% on CD8^+^ T cells, and from 37.9% to 2.3% on CD4^+^ T cells (Figure 2A). These varying percentages did not correlate with HIV viral loads, indicating variability in human immune responses among individual BLT mice with comparable HIV infections. As described in the following section, HIV-infected BLT mice were treated with either anti-PD-1 mAb at 13 weeks p.i., with control mAb, or received no further treatment. We hypothesized that PD-1 blockade would produce different effects in HIV-infected BLT mice with high versus low levels of CD8^+^ cells expressing PD-1. We therefore divided mice treated with anti-PD-1 mAb into 2 groups based on their PD-1^+^CD8^+^ levels: 1) a “PD1-LO” group with 3 mice with less than 30% PD-1^+^CD8^+^ cells, and 2) a “PD1-HI” group with 5 mice with more than 30% PD-1^+^CD8^+^ cells (Figure 2C). The percentage of CD4^+^ cells expressing PD-1 was not a factor in grouping the mice.

### *In vivo* anti-PD-1 mAb treatment decreased HIV viral load

**Figure 3:**
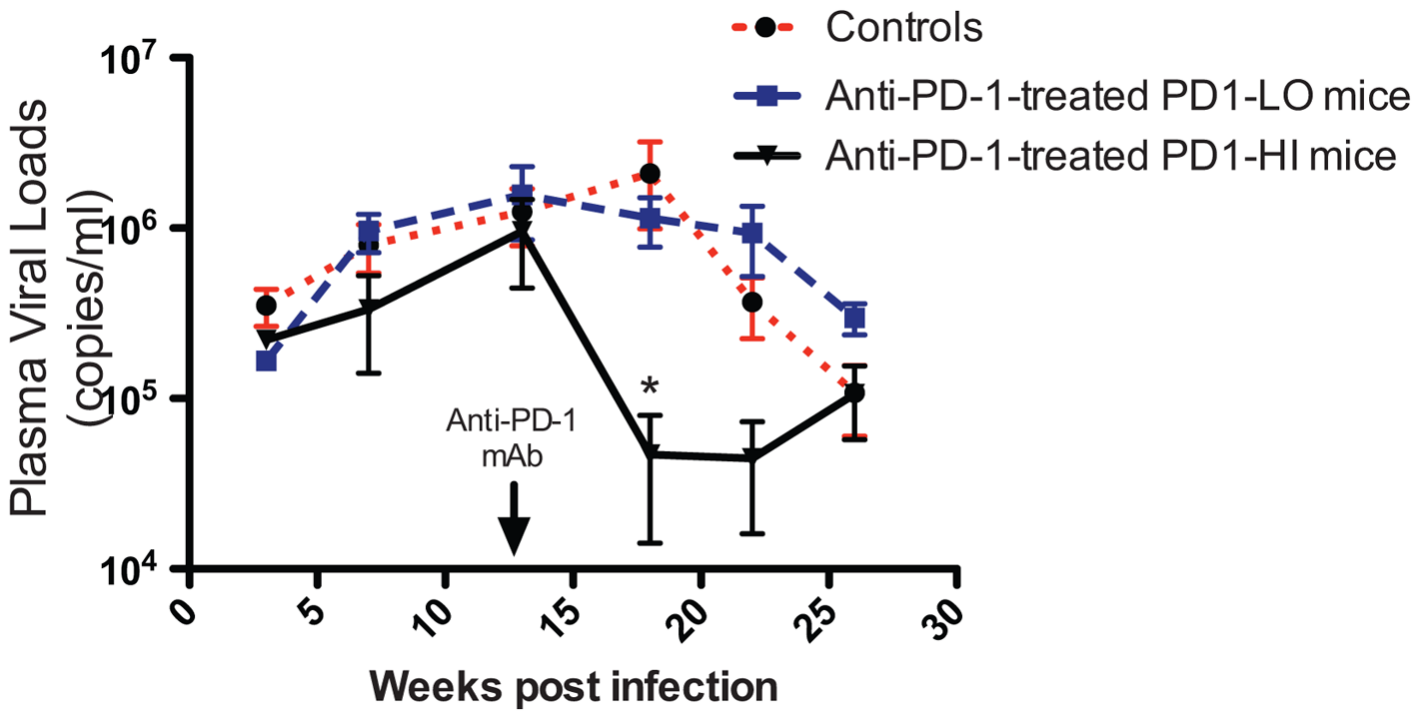
Effects of anti-PD-1 mAb or control treatment on HIV viral loads in chronically infected BLT mice. BLT mice infected with HIV-1 for 13 weeks were injected intraperitoneally with anti-PD-1 mAb, control mAb, or no Ab on days 0, 3, 7 and 10 (200 µg/dose, arrow). Peripheral blood was collected at multiple time points and HIV-1 plasma viral load was measured by quantitative RT-PCR. Graph represents mean viral load of Control (n = 10, control mAb or no Ab), anti-PD-1 mAb-treated PD1-LO mice (n = 3), and anti-PD-1 mAb-treated PD1-HI mice (n = 5). *P<0.05, Mann-Whitney test.

**Figure 4:**
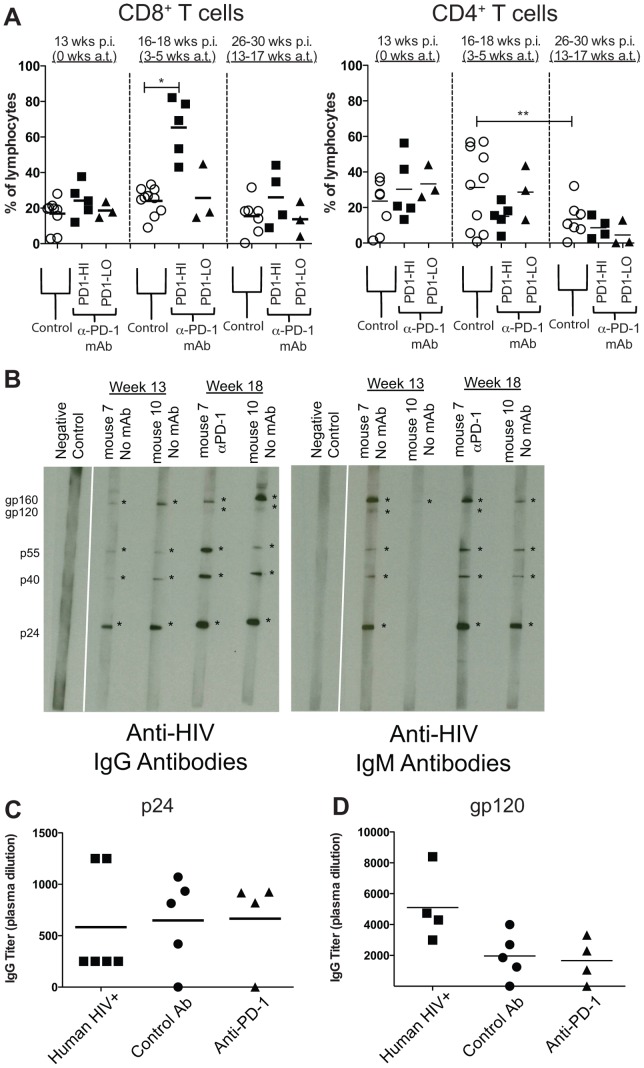
Cellular and humoral responses after anti-PD-1 mAb treatment. **A**) Percentages of human CD8^+^ and CD4^+^ T cells from lymphocyte gate at 13, 16–18 and 26–30 weeks post infection (p.i.), times corresponding to 0, 3–5 and 13–17 weeks after start of anti-PD-1 mAb treatment (a.t.), respectively. Horizontal lines within data points depict mean values. *P = 0.0007, Mann-Whitney test; **P = 0.047, Wilcoxon paired test. **B**) Western blot of plasma samples taken from 2 mice before and after anti-PD-1 mAb treatment showing anti-HIV IgG Abs and anti-HIV IgM Abs. *'s depict positive bands indicating the presence of antibodies to HIV proteins listed at left. Negative Controls, from the same blots as the data, were “cut-and-pasted” for image layout purposes. HIV-specific binding assays were performed using ELISA to measure IgG titers against **C**) p24 and **D**) gp120. Human plasma samples were collected 6-25 weeks post diagnosis of HIV-1 infection. BLT plasma samples were collected 26 weeks post infection.

PD-1 signaling was inhibited with a humanized mouse antibody specific to human PD-1 that blocks the interaction between PD-1 and its ligands (PD-L1 and PD-L2). Like the mAbs used in clinical trials of tumor immunotherapy [34], this antibody has an Fc that does not engage FcR or deplete PD-1^+^ cells. In order to determine if PD-1 blockade could lower HIV viral loads during the chronic phase of infection, BLT mice infected with HIV for at least 13 weeks (the time point where most CD8^+^ cells showed upregulation of PD-1) were treated with anti-PD-1 mAb. At 13 weeks p.i., the mean viral load of the 18 infected BLT mice was 1.3×10^6^ copies/mL (SEM = 3.1×10^5^). BLT mice in the Control group were either treated with isotype-matched control antibody (SYNAGIS) (n = 5) or did not receive any antibody treatment (n = 5). There were no significant differences in any parameter evaluated between mice receiving control mAb and mice receiving no Ab treatment. Therefore, mice receiving control or no Ab were analyzed together as a single control group. PD-1 blockade of the “PD1-LO” mice showed no significant change in their HIV viral loads from that of the Control mice (Figure 3). In contrast, PD-1 blockade of the “PD1-HI” mice dramatically decreased their HIV viral loads 5 weeks following the first dose of anti-PD-1 mAb by 1.7 logs (45-fold) compared to Controls, 1.4 logs (24-fold) compared to the “PD1-LO” group, and 1.4 logs (22-fold) compared to their pretreatment levels. The reduced viral loads in the “PD1-HI” group persisted for at least 9 weeks after the first treatment with anti-PD-1 mAb, after which the mean viral load started to increase at 13 weeks post treatment (Figure 3). This rise in viral loads 13 weeks following anti-PD-1 mAb treatment may reflect re-emergence of T cell exhaustion, emergence of viral escape mutations, or both. Declines in HIV viral loads were noted in the Control group at 22 and 26 weeks p.i., possibly due to declines in CD4^+^ T cell numbers, as shown in Figure 4a from 16-18 weeks to 26-30 weeks p.i. (*P* = 0.047), and previously noted by us at these times in other HIV-infected BLT mice [25].

### Anti-PD-1 mAb treatment affected T cells, but not B cell responses

Concurrent with viral load reductions, PD-1 blockade produced significant increases in the percentages of CD8^+^ T cells in the peripheral blood of chronically HIV-infected BLT mice which had high levels of CD8^+^PD-1^+^ expression. At 3 to 5 weeks following the initiation of anti-PD-1 mAb treatment, the percentage of CD8^+^ T cells in “PD1-HI” mice was 2.6-fold greater than in Control mice that were treated with control or no antibody (*P* = 0.0007), and 2.7-fold greater in the “PD1-HI” mice prior to anti-PD-1 treatment (Figure 4a). In contrast, PD-1 blockade produced no significant changes in the percentage of CD4^+^ T cells in the “PD1-HI” mice compared with Control HIV-infected mice (Figure 4a) or with “PD1-HI” mice prior to anti-PD-1 treatment. By 13–17 weeks following the initiation of anti-PD-1 antibody treatment, the increase in the percentage of CD8^+^ T cells in “PD1-HI” mice was no longer statistically significant compared with control HIV-infected mice (Figure 4a). The percentages of CD8^+^ or CD4^+^ T cells in “PD1-LO” mice were not significantly different from control HIV-infected mice or from the pre-mAb treatment levels in “PD1-LO” mice at either of the post-mAb treated time points evaluated (Figure 4a). The results suggest that PD-1 blockade produced a significant expansion of human CD8^+^ T cells, but not CD4^+^ T cells, in chronically HIV-infected BLT mice that had high levels of PD-1 expression on their CD8^+^ T cells.

PD-1 blockade did not affect the breadth or magnitude of anti-HIV antibody responses in chronically infected BLT mice. Western blots demonstrated both human IgM and IgG antibodies to multiple HIV antigens, including gp160, gp120, p65, p40, and p24, were generated in chronically infected mice (indicating that antibody class switching occurs in these animals, Figure 4b). However, anti-PD-1 mAb treatment of “PD1-HI” mouse did not increase the number of HIV antigens targeted compared to that treated with control Ab (Figure 4b). Anti-HIV Ab ELISAs demonstrated that anti-PD-1 mAb also did not increase antibody titers to those antigens that were targeted: IgG Ab titers to HIV p24 were found to be similar between anti-PD-1 mAb and control Ab-treated groups (Figure 4c), as were IgG titers to HIV envelope gp120 (Figure 4d).

### Co-expression of PD-1 with other inhibitory receptors differed between CD8^+^ and CD4^+^ T cells in chronically HIV-infected BLT mice

**Figure 5:**
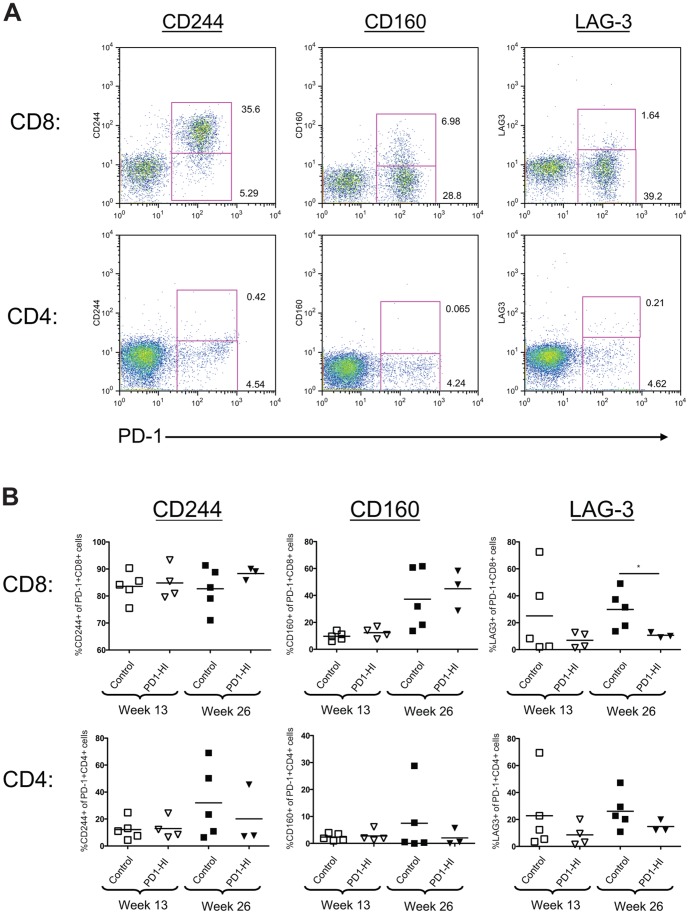
Co-expression of inhibitory receptors on CD8^+^ and CD4^+^ T cells in chronically HIV-infected BLT mice. **A**) Representative flow cytometry data of peripheral blood from an HIV-infected BLT mouse at 13 weeks post infection. Co-expression of CD244, CD160, and LAG-3 with PD-1 was determined on human CD8^+^ and CD4^+^ cells. **B**) Percentages of PD-1 expressing CD8^+^ and CD4^+^ T cells co-expressing CD244, CD160, and LAG-3 at 13 weeks and 26 weeks post infection. Horizontal lines within data points depict mean values. *P = 0.036, Mann-Whitney test.

In addition to PD-1, exhausted T cells may express a number of other inhibitory receptors during chronic infection, and co-expression of multiple inhibitory receptors has been associated with greater T cell exhaustion and more severe infection [35]. We consequently analyzed the co-expression of PD-1 with other inhibitory receptors associated with exhaustion, CD244, CD160, and LAG-3, by CD4^+^ and CD8^+^ cells in chronically HIV-infected BLT mice (Figure 5a). There were no significant differences in the percentages of PD-1-expressing CD8^+^ or CD4^+^ T cells that co-expressed CD244 or CD160 cells between the “PD1-HI” and Control mice before or after treatment with anti-PD-1, or control Ab, at 13 or 26 weeks p.i.(Figure 5b). Co-expression of LAG-3 by PD-1 expressing CD8+ T cells was significantly decreased following anti-PD-1 mAb treatment of “PD-HI” mice compared to Control mice at 26 weeks p.i. (*P* = 0.035).

There were marked differences in co-expression of PD-1 and these other inhibitor receptors between CD8^+^ and CD4^+^ T cells in both groups of mice, at both time points, however. An average of 84% of PD-1-expressing CD8^+^ T cells in mice in the “PD1-HI” and Control groups considered together co-expressed CD244 at 13 weeks p.i., whereas only 12.5% of the PD-1-expressing CD4^+^ cells of these mice did (*P* = 0.004, Figure 5b). This difference between CD8^+^ and CD4^+^ T cells in PD-1–CD244 co-expression persisted to 26 weeks p.i. (*P* = 0.008). PD-1–CD160 co-expression was also greater on the CD8^+^ than the CD4^+^ T cells of mice in the “PD1-HI” and control groups considered together, but only at the later time point of 26 weeks post infection. Few PD-1-expressing CD8^+^ or CD4^+^ T cells co-expressed CD160 at 13 weeks p.i., whereas at 26 weeks p.i. 40.1% of PD-1-expressing CD8^+^ T cells co-expressed CD160 compared with only 5.4% of PD-1-expressing CD4^+^ T cells (*P* = 0.008, Figure 5b). In contrast, there were no significant differences in PD-1–LAG-3 co-expression between CD8^+^ and CD4^+^ T cells in the chronically HIV-infected BLT mice at either time point (Figure 5b).

## Discussion

In this study, blockade of the PD-1-PD-L1 pathway using a short 10 day treatment with anti-PD-1 mAb expanded CD8^+^ T cells, and reduced viral loads in chronically HIV-infected BLT humanized mice by almost 2 logs, compared to control mice. These results extend our previous report that CD8^+^ T cell responses in acutely HIV-infected BLT mice resemble those in humans in terms of their specificity, kinetics, and immunodominance [31]. In acutely-infected BLT mice, we had previously demonstrated that the generation of HIV-specific CD8^+^ T cells was associated with selection of viral escape mutations in well-defined CD8 epitopes restricted by the human donor HLA alleles [31]. In this prior study, expression of the protective class I HLA allele B*57 by the human donor used to reconstitute BLT mice was associated with more sustained suppression of plasma viremia [31]. Taken together, these data suggest that the HIV-specific CD8^+^ T cells that expand in acutely infected BLT mice are functionally capable of limiting HIV replication. Our current study suggests that during more chronic HIV infection of BLT mice, CD8^+^ T cells become impaired, or “exhausted”, and that PD-1 blockade can reinvigorate these exhausted T cells to regain the capacity to limit HIV replication. This *in vivo* data is consistent with recent *in vitro* observations that mAbs to PD-1 and PD-L1 can augment HIV-specific CD8^+^ and CD4^+^ T cell proliferation and effector functions [15], [16], [17], [36].

Our study also validates recent *in vivo* demonstrations that inhibition of PD-1–PD-L1 signaling can reduce levels of SIV or HIV viremia in macaque [18], and humanized mouse [37], models of HIV respectively. Adding our results to those of Palmer and colleagues [37], inhibition of the PD-1–PD-L1 pathway now has been shown to reduce HIV viral loads in two different humanized mouse models, with targeting of PD-1 or its ligand PD-L1. Our study used BLT humanized mice, generated by surgically implanting fetal human thymic and liver tissues under the renal capsule of adult mice followed by adoptive transfer of autologous human fetal-derived CD34^+^ hematopoietic stem cells [25], [26], [27], whereas the study of Palmer *et al*. [37] used humanized BALB/c-Rag2^–/–^γc^–/–^ mice generated by the intrahepatic injection of human fetal liver-derived CD34^+^ hematopoietic stem cells into newborn mice [38]. In contrast to our use of anti-PD-1 mAb to inhibit PD-1–PD-L1 signaling, Palmer and colleagues used anti-PD-L1 mAb [37]. It will be of future interest to determine if there are different mechanisms involved between the two antibodies due to different target selection and antibody characteristics since PD-1 expression is more restricted than that of PD-L1. In addition, PD-L1 blockade would leave the PD-L2-PD-1 inhibitory pathway active whereas PD-1 blockade would leave the PD-L1-CD80 inhibitory pathway active. The ability of PD-1 or PD-L1 blockade to improve control of viremia in both of these humanized mouse models of chronic HIV infection underscores the therapeutic potential that PD-1–PD-L1 inhibition may have in human HIV infection, similar to its therapeutic potential in human cancer that has been demonstrated in recent clinical trials [34], [39], [40], [41].

CD8^+^ T cell expression of PD-1 did not increase uniformly in the chronically HIV-infected BLT mice in this study: most, but not all, or these mice demonstrated a dramatic increase in PD-1 expression on their CD8^+^ T cells. Prior to anti-PD-1 mAb treatment, HIV viral loads did not differ significantly between “PD1-HI” and “PD1-LO” groups of mice, suggesting that the differences in PD-1 expression between these mice did not result from differences in viral antigen burden. Rather, based on their differing responses to anti-PD-1 mAb treatment, we hypothesize that these mice differed in the quantities of HIV-specific CD8^+^ T cells that they generated prior to Ab treatment, with PD1-HI mice having a greater number of HIV-specific cells than PD1-LO mice. This hypothesis would be consistent with data from chronically HIV-infected humans, in whom significantly higher PD-1 levels are seen on total CD8^+^ T cells compared to HIV-seronegative patients, with the highest PD-1 expression found on tetramer^+^ HIV-specific CD8^+^ T cells [15]. This hypothesis would also be consistent with our observation of reduced HIV viral loads following anti-PD-1 treatment only in “PD1-HI” mice. Those mice generating higher amounts of HIV-specific CD8^+^ T cells would be expected first to have greater numbers of PD-1^+^ cells as those cells become functionally exhausted due to chronic exposure to viral antigens, and then to have more effective CD8^+^ T cell control of viremia as their greater numbers of HIV-specific but exhausted cells are reinvigorated by PD-1 blockade. This hypothesis would further be consistent with our observation of CD8^+^ T cell expansion following anti-PD-1 treatment only in “PD1-HI” mice. Those mice generating higher amounts of HIV-specific CD8^+^ T cells would also be expected to have greater numbers of cells that would proliferate in response to the high HIV antigen burden present at the time anti-PD-1 mAb treatment. To support this hypothesis, we tried to compare the numbers of HIV-specific CD8^+^ T cells that were present in the peripheral blood of “PD1-HI” versus “PD1-LO” mice at the time of anti-PD-1 mAb treatment. Our ability to detect these cells by standard interferon-γ ELISpot assays was precluded, however, by the failure of PBMCs from these mice, after being frozen and thawed, to generate interferon-γ.

HIV infection is associated with B cell dysfunction as well as T cell dysfunction [42], which has been attributed largely to bystander effects on B cells of immune activation driven by ongoing HIV replication [43]. PD-1 blockade in the SIV/macaque model significantly increased the titer of SIV-specific antibodies [18], suggesting the possibility that PD-1–PD-L1 signaling contributes to B cell dysfunction in HIV infection. PD-1 is upregulated on activated B cells [18], [44], and consequently could deliver negative signals to B cells as well as T cells. Alternatively, PD-1-induced B cell dysfunction could occur secondary to PD-1-induced dysfunction of T cell help. We consequently investigated whether PD-1 blockade could also increase the titer of HIV-specific antibodies in chronically HIV-infected BLT mice. Several prior studies of HIV infection in other humanized mouse models found little or no production of HIV-specific IgM or IgG antibodies post-infection [45], [46], [47]. In earlier work with the BLT mouse model, we showed that these mice can generate antibodies against a number of HIV antigens after infection, but did not analyze the isotypes of the antibodies produced [25]. Now we demonstrate that BLT mice can generate class-switched IgG antibodies against multiple HIV proteins, with titers approaching those of infected humans. In contrast to the SIV/macaque study cited above, however, we found PD-1 blockade produced no significant increases in the number of HIV antigens targeted, or in the titers of p24- or gp120-specific IgG antibodies, in chronically HIV-infected BLT mice. These results are consistent with a recent human study showing that titers and neutralizing activity of HIV-specific antibodies did not correlate with levels of PD-1expression on B cells in chronically infected subjects [44], suggesting that the immunological significance of PD-1 expression on B cells may be more important in SIV than HIV infection [43].

Given that exhausted T cells may express a number of other inhibitory receptors in addition to PD-1 during chronic infection, and that co-expression of multiple inhibitory receptors has been associated with greater T cell exhaustion [35], we assessed the co-expression of three of these other inhibitory receptors, CD244, CD160, and LAG-3, with PD-1 in chronically HIV-infected mice. In chronically HIV-infected humans, virus-specific CD8^+^ T cells have recently been noted to have substantial co-expression of CD244 and CD160 with PD-1, but little of LAG-3 [36]. Consistent with these results, we found that in the chronically HIV-infected BLT mice, the majority of CD8^+^ T cells co-expressed PD-1 and CD244, and a substantial minority co-expressed PD-1 and CD160, whereas few cells co-expressed PD-1 and LAG-3. In contrast to the CD8^+^ T cells, none of these other three inhibitory receptors were substantially co-expressed by PD-1 expressing CD4^+^ T cells in the chronically infected BLT mice. As blocking both PD-1 and LAG-3 in chronic LCMV infection in mice has been shown to have additive therapeutic benefits [35], it will be of interest to determine if blocking CD244 or CD160 could further improve the control of HIV seen in chronically HIV-infected humanized mice with inhibition of their PD-1–PD-L signaling.

The results from this study demonstrate that *in vivo* blockade of PD-1 during chronic HIV infection can produce significant expansions of CD8^+^ T cells and decreases in viral loads. These positive effects of antibodies blocking the PD-1-PD-L1 pathway in humanized mice further indicate that reinvigoration of exhausted T cells has the potential to be a novel therapeutic approach to chronic HIV infection, as suggested by studies performed with human T cells *ex vivo* and with SIV in macaques *in vivo*. Our results, together with those of Palmer and colleagues [37], also suggest that the new generation of humanized mouse models, with their improved capacity to model the human immune system, now appear capable of evaluating novel immunomodulatory approaches to HIV infection in humans.

## References

[1] Borrow P, Lewicki H, Hahn BH, Shaw GM, Oldstone MB (1994) Virus-specific CD8+ cytotoxic T-lymphocyte activity associated with control of viremia in primary human immunodeficiency virus type 1 infection. J Virol 68: 6103–6110. 8057491 10.1128/jvi.68.9.6103-6110.1994 PMC237022

[2] Koup RA, Safrit JT, Cao Y, Andrews CA, McLeod G, et al (1994) Temporal association of cellular immune responses with the initial control of viremia in primary human immunodeficiency virus type 1 syndrome. J Virol 68: 4650–4655. 8207839 10.1128/jvi.68.7.4650-4655.1994 PMC236393

[3] Pantaleo G, Demarest JF, Soudeyns H, Graziosi C, Denis F, et al (1994) Major expansion of CD8+ T cells with a predominant V beta usage during the primary immune response to HIV. Nature 370: 463–467. 8047166 10.1038/370463a0

[4] Walker CM, Moody DJ, Stites DP, Levy JA (1986) CD8+ lymphocytes can control HIV infection in vitro by suppressing virus replication. Science 234: 1563–1566. 2431484 10.1126/science.2431484

[5] Schmitz JE, Kuroda MJ, Santra S, Sasseville VG, Simon MA, et al (1999) Control of viremia in simian immunodeficiency virus infection by CD8+ lymphocytes. Science 283: 857–860. 9933172 10.1126/science.283.5403.857

[6] Yang OO, Kalams SA, Rosenzweig M, Trocha A, Jones N, et al (1996) Efficient lysis of human immunodeficiency virus type 1-infected cells by cytotoxic T lymphocytes. J Virol 70: 5799–5806. 8709196 10.1128/jvi.70.9.5799-5806.1996 PMC190594

[7] Yang OO, Garcia-Zepeda EA, Walker BD, Luster AD (2002) Monocyte chemoattractant protein-2 (CC chemokine ligand 8) inhibits replication of human immunodeficiency virus type 1 via CC chemokine receptor 5. J Infect Dis 185: 1174–1178. 11930329 10.1086/339678

[8] Yang OO, Swanberg SL, Lu Z, Dziejman M, McCoy J, et al (1999) Enhanced inhibition of human immunodeficiency virus type 1 by Met-stromal-derived factor 1beta correlates with down-modulation of CXCR4. J Virol 73: 4582–4589. 10233917 10.1128/jvi.73.6.4582-4589.1999 PMC112499

[9] Wagner L, Yang OO, Garcia-Zepeda EA, Ge Y, Kalams SA, et al (1998) Beta-chemokines are released from HIV-1-specific cytolytic T-cell granules complexed to proteoglycans. Nature 391: 908–911. 9495345 10.1038/36129

[10] Yang OO, Kalams SA, Trocha A, Cao H, Luster A, et al (1997) Suppression of human immunodeficiency virus type 1 replication by CD8+ cells: evidence for HLA class I-restricted triggering of cytolytic and noncytolytic mechanisms. J Virol 71: 3120–3128. 9060675 10.1128/jvi.71.4.3120-3128.1997 PMC191444

[11] Cocchi F, DeVico AL, Garzino-Demo A, Arya SK, Gallo RC, et al (1995) Identification of RANTES, MIP-1 alpha, and MIP-1 beta as the major HIV-suppressive factors produced by CD8+ T cells. Science 270: 1811–1815. 8525373 10.1126/science.270.5243.1811

[12] Zajac AJ, Blattman JN, Murali-Krishna K, Sourdive DJ, Suresh M, et al (1998) Viral immune evasion due to persistence of activated T cells without effector function. J Exp Med 188: 2205–2213. 9858507 10.1084/jem.188.12.2205 PMC2212420

[13] Wherry EJ, Ahmed R (2004) Memory CD8 T-cell differentiation during viral infection. J Virol 78: 5535–5545. 15140950 10.1128/JVI.78.11.5535-5545.2004 PMC415833

[14] Barber DL, Wherry EJ, Masopust D, Zhu B, Allison JP, et al (2006) Restoring function in exhausted CD8 T cells during chronic viral infection. Nature 439: 682–687. 16382236 10.1038/nature04444

[15] Day CL, Kaufmann DE, Kiepiela P, Brown JA, Moodley ES, et al (2006) PD-1 expression on HIV-specific T cells is associated with T-cell exhaustion and disease progression. Nature 443: 350–354. 16921384 10.1038/nature05115

[16] Petrovas C, Casazza JP, Brenchley JM, Price DA, Gostick E, et al (2006) PD-1 is a regulator of virus-specific CD8+ T cell survival in HIV infection. J Exp Med 203: 2281–2292. 16954372 10.1084/jem.20061496 PMC2118095

[17] Trautmann L, Janbazian L, Chomont N, Said EA, Gimmig S, et al (2006) Upregulation of PD-1 expression on HIV-specific CD8+ T cells leads to reversible immune dysfunction. Nat Med 12: 1198–1202. 16917489 10.1038/nm1482

[18] Velu V, Titanji K, Zhu B, Husain S, Pladevega A, et al (2009) Enhancing SIV-specific immunity in vivo by PD-1 blockade. Nature 458: 206–210. 19078956 10.1038/nature07662 PMC2753387

[19] Akkina R (2013) New generation humanized mice for virus research: comparative aspects and future prospects. Virology 435: 14–28. 23217612 10.1016/j.virol.2012.10.007 PMC3932328

[20] Mosier DE, Gulizia RJ, Baird SM, Wilson DB (1988) Transfer of a functional human immune system to mice with severe combined immunodeficiency. Nature 335: 256–259. 2970594 10.1038/335256a0

[21] McCune JM, Namikawa R, Kaneshima H, Shultz LD, Lieberman M, et al (1988) The SCID-hu mouse: murine model for the analysis of human hematolymphoid differentiation and function. Science 241: 1632–1639. 2971269 10.1126/science.241.4873.1632

[22] Mosier DE, Gulizia RJ, Baird SM, Wilson DB, Spector DH, et al (1991) Human immunodeficiency virus infection of human-PBL-SCID mice. Science 251: 791–794. 1990441 10.1126/science.1990441

[23] Namikawa R, Kaneshima H, Lieberman M, Weissman IL, McCune JM (1988) Infection of the SCID-hu mouse by HIV-1. Science 242: 1684–1686. 3201256 10.1126/science.3201256

[24] Shultz LD, Brehm MA, Garcia-Martinez JV, Greiner DL (2012) Humanized mice for immune system investigation: progress, promise and challenges. Nat Rev Immunol 12: 786–798. 23059428 10.1038/nri3311 PMC3749872

[25] Brainard DM, Seung E, Frahm N, Cariappa A, Bailey CC, et al (2009) Induction of robust cellular and humoral virus-specific adaptive immune responses in human immunodeficiency virus-infected humanized BLT mice. J Virol 83: 7305–7321. 19420076 10.1128/JVI.02207-08 PMC2704767

[26] Melkus MW, Estes JD, Padgett-Thomas A, Gatlin J, Denton PW, et al (2006) Humanized mice mount specific adaptive and innate immune responses to EBV and TSST-1. Nat Med 12: 1316–1322. 17057712 10.1038/nm1431

[27] Lan P, Tonomura N, Shimizu A, Wang S, Yang YG (2006) Reconstitution of a functional human immune system in immunodeficient mice through combined human fetal thymus/liver and CD34+ cell transplantation. Blood 108: 487–492. 16410443 10.1182/blood-2005-11-4388

[28] Denton PW, Estes JD, Sun Z, Othieno FA, Wei BL, et al (2008) Antiretroviral pre-exposure prophylaxis prevents vaginal transmission of HIV-1 in humanized BLT mice. PLoS Med 5: e16. 18198941 10.1371/journal.pmed.0050016 PMC2194746

[29] Sun Z, Denton PW, Estes JD, Othieno FA, Wei BL, et al (2007) Intrarectal transmission, systemic infection, and CD4+ T cell depletion in humanized mice infected with HIV-1. J Exp Med 204: 705–714. 17389241 10.1084/jem.20062411 PMC2118553

[30] Tonomura N, Habiro K, Shimizu A, Sykes M, Yang YG (2008) Antigen-specific human T-cell responses and T cell-dependent production of human antibodies in a humanized mouse model. Blood 111: 4293–4296. 18270327 10.1182/blood-2007-11-121319 PMC2288728

[31] Dudek TE, No DC, Seung E, Vrbanac VD, Fadda L, et al (2012) Rapid evolution of HIV-1 to functional CD8(+) T cell responses in humanized BLT mice. Sci Transl Med 4: 143ra198. 10.1126/scitranslmed.3003984 PMC3685142 22814851

[32] Boutwell CL, Rowley CF, Essex M (2009) Reduced viral replication capacity of human immunodeficiency virus type 1 subtype C caused by cytotoxic-T-lymphocyte escape mutations in HLA-B57 epitopes of capsid protein. J Virol 83: 2460–2468. 19109381 10.1128/JVI.01970-08 PMC2648284

[33] Dorfman DM, Brown JA, Shahsafaei A, Freeman GJ (2006) Programmed death-1 (PD-1) is a marker of germinal center-associated T cells and angioimmunoblastic T-cell lymphoma. Am J Surg Pathol 30: 802–810. 16819321 10.1097/01.pas.0000209855.28282.ce PMC3137919

[34] Topalian SL, Hodi FS, Brahmer JR, Gettinger SN, Smith DC, et al (2012) Safety, activity, and immune correlates of anti-PD-1 antibody in cancer. N Engl J Med 366: 2443–2454. 22658127 10.1056/NEJMoa1200690 PMC3544539

[35] Blackburn SD, Shin H, Haining WN, Zou T, Workman CJ, et al (2009) Coregulation of CD8+ T cell exhaustion by multiple inhibitory receptors during chronic viral infection. Nat Immunol 10: 29–37. 19043418 10.1038/ni.1679 PMC2605166

[36] Porichis F, Kwon DS, Zupkosky J, Tighe DP, McMullen A, et al (2011) Responsiveness of HIV-specific CD4 T cells to PD-1 blockade. Blood 118: 965–974. 21652684 10.1182/blood-2010-12-328070 PMC3148173

[37] Palmer BE, Neff CP, Lecureux J, Ehler A, Dsouza M, et al (2013) In vivo blockade of the PD-1 receptor suppresses HIV-1 viral loads and improves CD4+ T cell levels in humanized mice. J Immunol 190: 211–219. 23209326 10.4049/jimmunol.1201108 PMC3529847

[38] Berges BK, Wheat WH, Palmer BE, Connick E, Akkina R (2006) HIV-1 infection and CD4 T cell depletion in the humanized Rag2-/-gamma c-/- (RAG-hu) mouse model. Retrovirology 3: 76. 17078891 10.1186/1742-4690-3-76 PMC1635423

[39] Wolchok JD, Kluger H, Callahan MK, Postow MA, Rizvi NA, et al (2013) Nivolumab plus Ipilimumab in Advanced Melanoma. N Engl J Med 369: 122–133. 23724867 10.1056/NEJMoa1302369 PMC5698004

[40] Hamid O, Robert C, Daud A, Hodi FS, Hwu WJ, et al (2013) Safety and Tumor Responses with Lambrolizumab (Anti-PD-1) in Melanoma. N Engl J Med 369: 134–144. 23724846 10.1056/NEJMoa1305133 PMC4126516

[41] Brahmer JR, Tykodi SS, Chow LQ, Hwu WJ, Topalian SL, et al (2012) Safety and activity of anti-PD-L1 antibody in patients with advanced cancer. N Engl J Med 366: 2455–2465. 22658128 10.1056/NEJMoa1200694 PMC3563263

[42] Moir S, Fauci AS (2008) Pathogenic mechanisms of B-lymphocyte dysfunction in HIV disease. J Allergy Clin Immunol 122: : 12–19; quiz 20–11. 10.1016/j.jaci.2008.04.034 PMC2708937 18547629

[43] Moir S, Fauci AS (2013) Insights into B cells and HIV-specific B-cell responses in HIV-infected individuals. Immunol Rev 254: 207–224. 23772622 10.1111/imr.12067

[44] Boliar S, Murphy MK, Tran TC, Carnathan DG, Armstrong WS, et al (2012) B-lymphocyte dysfunction in chronic HIV-1 infection does not prevent cross-clade neutralization breadth. J Virol 86: 8031–8040. 22623771 10.1128/JVI.00771-12 PMC3421653

[45] Gorantla S, Sneller H, Walters L, Sharp JG, Pirruccello SJ, et al (2007) Human immunodeficiency virus type 1 pathobiology studied in humanized BALB/c-Rag2-/-gammac-/- mice. J Virol 81: 2700–2712. 17182671 10.1128/JVI.02010-06 PMC1865995

[46] Baenziger S, Tussiwand R, Schlaepfer E, Mazzucchelli L, Heikenwalder M, et al (2006) Disseminated and sustained HIV infection in CD34+ cord blood cell-transplanted Rag2-/-gamma c-/- mice. Proc Natl Acad Sci U S A 103: 15951–15956. 17038503 10.1073/pnas.0604493103 PMC1635108

[47] Berges BK, Rowan MR (2011) The utility of the new generation of humanized mice to study HIV-1 infection: transmission, prevention, pathogenesis, and treatment. Retrovirology 8: 65. 21835012 10.1186/1742-4690-8-65 PMC3170263

